# Symposium report: breast cancer in India—trends, environmental exposures and clinical implications

**DOI:** 10.1007/s10552-021-01428-y

**Published:** 2021-04-28

**Authors:** Jasmine A. McDonald, Roshni Rao, Marley Gibbons, Rajiv Janardhanan, Surinder Jaswal, Ravi Mehrotra, Manoj Pandey, Venkatraman Radhakrishnan, Pooja Ramakant, Nandini Verma, Mary Beth Terry

**Affiliations:** 1 Department of Epidemiology, Mailman School of Public Health, Columbia University Irving Medical Center, 722 West 168th St, New York, NY 10032 USA; 2 Herbert Irving Comprehensive Cancer Center, Columbia University Irving Medical Center, New York, NY USA; 3 Division of Breast, Melanoma and Soft Tissue Surgery, Columbia University Vagelos College of Physicians and Surgeons, Columbia University Irving Medical Center, New York, NY USA; 4 Laboratory of Disease Dynamics & Molecular Epidemiology, Amity Institute of Public Health, Amity University, Uttar Pradesh, India; 5 School of Research Methodology, Centre for Health and Mental Health, School of Social Work, Tata Institute of Social Sciences, Mumbai, Maharashtra India; 6 Indian Council of Medical Research (ICMR) - India Cancer Research Consortium, New Delhi, India; 7 Institute of Medical Sciences, Department of Surgical Oncology, Banaras Hindu University, Varanasi, Uttar Pradesh India; 8 Cancer Institute (W.I.A), Chennai, India; 9 King Georges Medical University, Lucknow, Uttar Pradesh India; 10 TNBC Precision Medicine Research Laboratory, Advanced Centre for Treatment, Research, and Education in Cancer, Tata Memorial Center, Khargar, Navi Mumbai, Maharashtra India; 11 Homi Bhabha National Institute, Training School Complex, Anushakti Nagar, Mumbai, Maharashtra India

**Keywords:** India, Breast cancer, Triple-negative breast cancer, Risk factors, Incidence trends

## Abstract

**Purpose:**

Incidence of breast cancer (BC), particularly in young women, are rising in India. Without population-based mammography screening, rising rates cannot be attributed to screening. Investigations are needed to understand the potential drivers of this trend.

**Methods:**

An international team of experts convened to discuss the trends, environmental exposures, and clinical implications associated with BC in India and outlined recommendations for its management.

**Results:**

Panels were structured across three major BC themes (*n* = 10 presentations). The symposium concluded with a semi-structured Think Tank designed to elicit short-term and long-term goals that could address the challenges of BC in India.

**Conclusion:**

There was consensus that the prevalence of late-stage BC and the high BC mortality rates are associated with the practice of detection, which is primarily through clinical and self-breast exams, as opposed to mammography. Triple-Negative BC (TNBC) was extensively discussed, including TNBC etiology and potential risk factors, the limited treatment options, and if reported TNBC rates are supported by rigorous scientific evidence. The Think Tank session yielded long-term and short-term goals to further BC reduction in India and included more regional etiological studies on environmental exposures using existing India-based cohorts and case–control studies, standardization for molecular subtyping of BC cases, and improving the public’s awareness of breast health.

## Introduction

In India, breast cancer (BC) has emerged as the second most common type of malignancy within the past 25 years. In terms of disability-adjusted life years (DALYs), where BC used to be less prevalent than the more prevalent stomach, cervical, and leukemia diagnoses, according to data accumulated up until 2016, BC is surpassed only by cervical cancer [1]. Moreover, India has one of the highest rates of the most aggressive subtype of BC referred to as Triple-Negative Breast Cancer (TNBC) [2, 3]. The rates of TNBC in India are almost double that of the United States (US) with estimates as high as 28% to one-third of all BC in India compared to 12–15% in the US [4]. Characterized by the lack of estrogen, progesterone, and HER2Neu receptors, TNBC lacks treatment options for targeted therapy and has a higher fatality rate as compared to other BC subtypes which tends to occur in younger patients (women under age 50) and women of African descent [5].

**Fig. 1:**
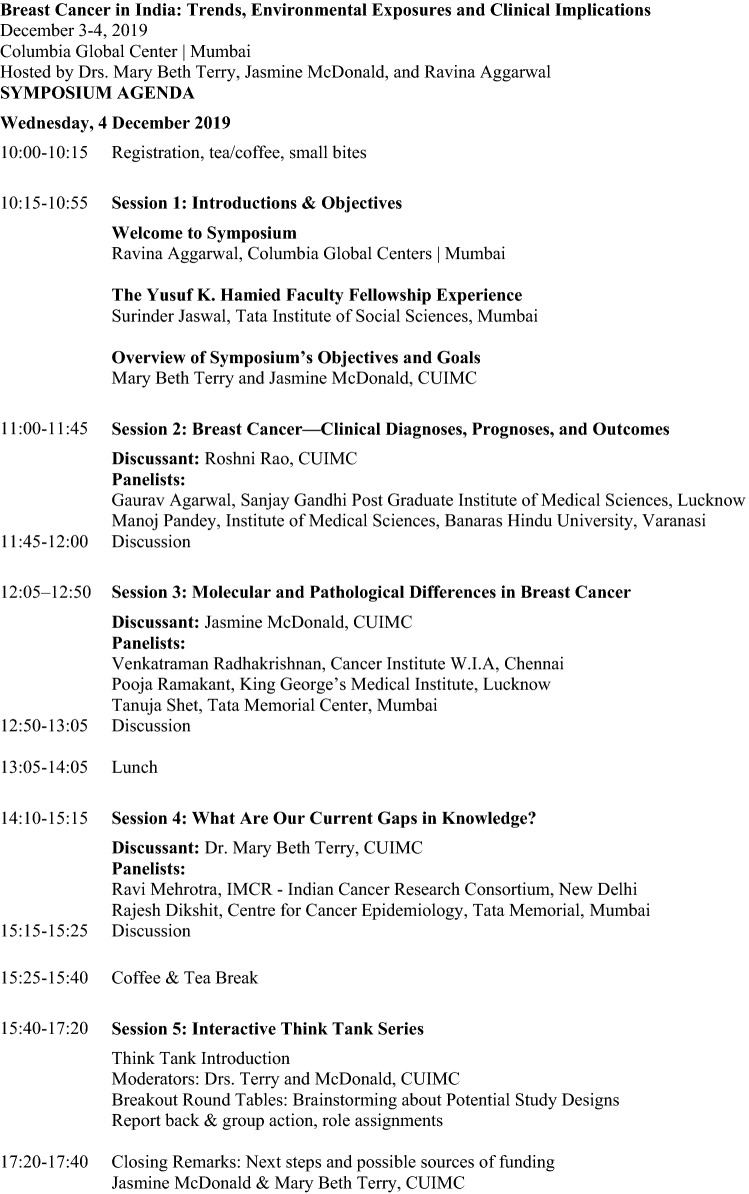
.

With the goal of employing collaborative efforts at reducing BC incidence and mortality in India, a group of cancer experts and key stakeholders were invited to the *Breast Cancer in India: Trends, Environmental Exposures and Clinical Implications* symposium held at the Columbia Global Centers | Mumbai on 4 December 2019 (see Fig. 1). The three objectives for this meeting were as follows: (1) gather cancer experts and key stakeholders to discuss the challenges and research opportunities to reduce BC in India, (2) consider etiological and potential exposures that may contribute to the rates of early-onset BC, and (3) develop short- and long-term collaborative research and training goals for BC risk reduction.

In this report, we summarize the interdisciplinary discussions from experts across relevant fields that led to the groups’ research, policy and programming, and advocacy recommendations that are needed to address BC in India, particularly for women under 50 years.

## Breast cancer—clinical diagnoses, prognoses, and outcomes

Panelists focused on identifying temporal trends in BC, delineating actual rates of TNBC, and discussing current obstacles to BC care in India.

### Younger age

There was general agreement that BC in India is more often seen in a younger population when compared to Western cohorts. Data from Banaras Hindu University (BHU) reveal a mean age of 51, and data from the Sanjay Gandhi Postgraduate Institute of Medical Sciences (SGPGIMS) in Lucknow, India reports a mean age of 49.7 years. Data from the Indian Council of Medical Research between 1982 and 2005 observed an annual percent change (APC) as high as 4.2% in Nagpur among women age 15–34 [6]. The group discussed whether the younger age was driven by higher age-specific incidence rates in India or related to biases based on clinical presentation and who is treated and can travel to cancer hospitals may be related to younger ages.

### Higher stage

Another significant issue is the stage of presentation. A cohort of 3,473 patients from SGPGIMS demonstrated an 11% incidence of Stage IV disease at presentation, and although this did decrease over time from 1990 when the incidence of Stage IV disease was 20%, this is significantly higher than the 6% incidence observed in the US [7]. The group at BHU reported a 35% incidence of Stage IV BC at initial presentation. However, given this is not population-based data but hospital-based data from an institution with tertiary care center status, “referral bias” is an issue.

There was no consensus on what screening modality would best serve the Indian population to downshift the stage of presentation. Given the known limitations of mammography in dense breast tissue and in young women, attendees agreed that population-based mammography would not be optimal as a population-based approach for screening particularly given the earlier age of onset in Indian women [8]. The value of ultrasound screening requires further investigation and there are concerns about overwhelming the health care infrastructure with false positives. It is unclear whether clinical breast examination alone is sufficient, and the challenge would be determining who should perform this and what training level is required to maximize benefit. Another key recommendation would be to identify optimal screening avenues (and community initiatives with the help of local primary care workers) for the Indian population.

#### TNBC

Single-institution data support higher rates of TNBC in the Indian population. The BHU group reports a 32% (*n* = 113/349) rate of TNBC and SGPGIMS data indicate a 29% (*n* = 1,042/3,473) incidence of TNBC. These are both higher than what is observed in Western populations, although some research indicates the oncologic outcomes in the Indian population may not be influenced as heavily by hormone status [9]. This finding may be related to the overall lower survival from BC when it presents with advanced disease. The recommendation from the group includes the standardization of pathologic reporting and analysis (outlined in the following sections), as well as consideration for a national TNBC registry to prospectively collect national level data that can also underline the incidence of BC by state to understand regional epidemiology.

## Molecular and pathological differences in breast cancer

### Debate: TNBC rates

Are TNBC rates in India rising? As noted above, India-based TNBC data are limited by the lack of population-based rates [4, 10]; nonetheless, some experts suggest that TNBC incidences are rising. A recent meta-analysis across 17 cross-sectional studies found a 31% prevalence of TNBC in India with study heterogeneity not explained by available study-level characteristics (i.e., age, menopausal status, grade, tumor size). This high prevalence is observed in women of African descent, with population-based studies observing between 21 and 46% among Black women in the United States and 20% to 82% in African women [11]. Additional factors for TNBC include younger age, BRCA1 mutation status, higher body mass index, and a host of reproductive factors, including earlier age at menarche, younger age at first full-term birth, higher parity, and shorter duration of breastfeeding. How these risk factors distribute across Indian women with TNBC is of interest.

Several suggested differences in TNBC rates were driven by the distribution of tumors observed in India compared to countries that have population-based screening and therefore a larger proportion of smaller tumors [12]. TNBC rates may also not be as high as estimates suggest as standards related to core biopsy collection, processing, and receptor assessments are not consistent across India [13]. While some hospitals have the resources to adequately prepare the core biopsy samples and implement standardized ER/PR and HER2 testing with routine quality checks to ensure standards of assessments are met, some hospitals are lacking these practices or the access to quality molecular testing. This possible faulty testing could result in an over-estimate. However, it is also possible that as these standardizations are put in place, while performance will improve, the proportions of TNBC may remain constant, which would suggest that TNBC rates are not changing.

### Improving TNBC classification and risk stratification

TNBC is a heterogeneous disease where the majority are basal-like subtype, but a TNBC diagnosis requires confirmation by gene expression signatures and immunohistochemistry analysis [12], which experts agreed could present significant methodological challenges. Challenges in subtyping lead to challenges in identifying novel treatments and more effectively targeted drugs [14]. For example, attempts to reproduce certain TNBC subtypes have been inconsistent and therefore researchers are unable to validate gene signatures predictive of disease-free survival [14]. Overall, more research is needed on these aggressive tumors, including examination of precursor lesions for TNBC and the exploration of possible causal associations between infectious agents (e.g., EBV, microbiome, etc.) and TNBC. Of universal concern was that the majority of BC cases in India are late stage which results in poorer BC prognoses. However, with limited effective treatment options for TNBC, the drugs that are available do little to improve cancer prognoses [15, 16]. To improve prognoses, women need to be observed at earlier stages. A step toward this would be implementing risk prediction modeling. For example, some of the genes that are associated with TNBC are also genes that might be able to be screened for across different high-risk populations [17].

## What are our current gaps in knowledge?

In addition to greater standardization in molecular subtyping, there is also a pressing need to expand etiological research into what may be the key drivers of the increase in BC rates. As population-based screening mammography is not utilized in India, increased screening is not an explanation for the rising incidence trends.

### *Geographic variation*: *what are the drivers?*

In addition to the increasing trends throughout India, there remains substantial geographic variation in BC incidence across India from a high of 240.8 DALY rate in Kerala to a low of 58.4 DALY rate in Sikkim [1]. Importantly, not only is there substantial variation in DALY rates across India but also within areas of similar epidemiological transition levels [9]. The great variability across and within regions supports that there is a need to understand the drivers of these differences, including difference in demographic characteristics and access to resources across regions/states, particularly in relation to class and caste and rural, tribal, and urban settings.

There is growing evidence that established risk factors for BC, including genetic variants in BC susceptibility genes, are also important predictors of BC risk in India. For example, in a large case–control study with over 1,600 cases and 1,500 controls conducted in Mumbai India, Dr. Dikshit and colleagues reported that central adiposity (measured by a high waist to hip ratio) was associated with both pre- and postmenopausal BC [18]. His team also reported that 11 genetic variants from eight genomic regions (*FGFR2, 9q31.2, MAP3K, CCND1, ZM1Z1, RAD51L11, ESR1,* and *UST*) were also associated with BC cases and controls from Mumbai, India, and this study also reported an increased prevalence of a number of estrogen metabolizing genes in cases compared to controls [19]. Of note, although many established BC risk factors, like central adiposity and late age at first pregnancy in rural and urban areas of India, the prevalence of these risk factors vary across rural and urban settings [20].

At the symposium, discussion about the role of early life exposures, including risk factors that may be related to growth and breast development and windows of susceptibility in driving BC trends [21], was supported by recent data presented by Dr. Dikshit showing that women who spent the first 20 years of life in rural settings compared to women who spent their first 20 years of life in urban settings had a lower breast cancer risk (personal communication). There was universal agreement that more etiologic research was needed particularly on environmental exposures of air pollution and polycyclic aromatic hydrocarbons (PAHs), and other environmental pollutants and endocrine disrupting chemicals (EDCs). EDCs interfere with various aspects of metabolism and is also a risk factor for metabolic diseases, such as obesity, which is indeed a risk factor strongly associated with aggressive breast cancer lesions. Participants discussed that sustained human exposure to these chemicals may have a disrupting role in the functioning of the endocrine/hormonal system and metabolic pathways [21, 22] and the need for additional studies to be conducted in India.

### Improving existing infrastructure

The panelists reviewed screening recommendations already established through governmental public health organizations, including (1) clinical breast examination combined with diagnostic ultrasound; (2) awareness and education supplemented with clinical breast examination screening and early detection programs that should bring women in for examination at least once every 3 years; and (3) fine needle aspiration cytological tests or core needle biopsy with appropriate follow-up services should be made available to provide patient access to prompt diagnosis and treatment [23]. Dr. Ravi Mehrotra, Director of the India Cancer Research Consortium, outlined existing mechanisms and coverage of BC screening through integration and training of health care providers from many different levels of the society, including village health care workers, nurses, dentists, gynecologists, surgeons, and radiologists [24].

Thus, the major gaps in knowledge called for (1) more research examining environmental exposures and additional studies of early life exposures; (2) implementation of scientific methods to identify ways to increase clinical breast exams as well as increasing awareness of BC risk and BC risk factors; and (3) evaluation of screening strategies to reduce the burden of late-stage diagnoses.

## Suggestions and recommendations

The Think Tank portion of the symposium was a facilitator-led semi-structured brainstorming intended to discuss some of the short-term and long-term goals that could address BC in India. Throughout the discussion, attendees referred to the Pulse Polio and National AIDS Control Programme (NACP) as successful campaigns we could learn from and possibly build upon in order to implement elements of the recommendations. During the session, attendees gathered in smaller pre-assigned groups to develop recommendations for *short- and long-term research strategies for primary prevention* and for *short- and long-term solutions focused on clinical outcomes*. The “best picks” were reported to the group followed by brief discussion. In closing, each attendee shared personal thoughts on what would be the most pressing strategy for addressing the burden of early-onset BC in India. The recommendations fell into three actionable elements: Research, Policy/Programming, and Advocacy (Table 1).

**Table 1:** Symposium recommendations related to short- and long-term strategies to address breast cancer in India

Suggested ways forward	Research	Policy/programming	Advocacy
Increase female body awareness and reduce stigma associated with breast cancer (BC)	Perform a case study of regionally successful public health campaigns (eg. Pulse Polio Campaign,^a^ National AIDS Control Programme)^b^	Encourage trained medical personnel to perform clinical breast examination	Enlist local champions to gather local explanation models for BC symptoms, fears, common misconceptions, slang, and vernacular around breast cancer Institute a nation-wide campaign to encourage breast health Design a mobile app to disseminate validated information on cancer risk and diagnosis. Features include: - Interactive map of treatment resources throughout India - Confidential channel for women to privately submit health-related questions
		Create a safety net of health centers with trained health workers and navigators knowledgeable about next steps for a suspected diagnosis of BC	
Examine the relationship between lifestyle factors at critical points across the life course and the risk of developing early-onset BC	Longitudinal study of diet to examine the correlation between weight gain at key points across the life course (e.g., menarche, menopause) and risk of early-onset BC Studies of environmental exposures during windows of BC susceptibility	Target critical periods across the life course for clinical breast examination to improve detection and prevention of BC	Elevate awareness of maintaining a healthy lifestyle for BC risk reduction. Including the emphasis on physical activity guidelines and other behavioral measures for reducing obesity
Address early-onset BC	Create a prospective BC database to inform predictive scoring system for BC risk and treatment	Apply a predictive scoring system to identify patients at high risk for early-onset BC and inform standardization of treatment with the involvement and collaboration of national organizations like the National Cancer Grid ^c^	Encourage women to have a routine clinical breast exam to improve BC detection
Examine the relationship between environmental exposures and the development of early-onset BC	Identify which regions of India BC referral cases are coming from using the Population-Based Cancer Registry data Measure regionally, environmental factors that could influence BC susceptibility Conduct molecular epidemiologic studies	Reduce indoor and outdoor pollution Implement agricultural standards for farmers and farm workers, especially pregnant women, to wear protective gear	
Understand the molecular signature of triple-negative breast cancer (TNBC)	Explore if there is a pathogenic role in the development of TNBC precursor legions and the immune response for TNBC cases Conduct a clinical trial to explore whether dose-dense chemotherapy improves neoadjuvant treatment outcomes	Create a national TNBC Registry and incentivize clinicians to submit all TNBC cases. Database can be used to characterize differences in TNBC incidence across states and regions	
Create a clinical trial to improve neoadjuvant treatment outcomes			
Standardize, expand and enforce a uniform standard of care for BC	Reexamine existing standards of care for BC to ensure they include standards for well-recorded follow-up appointments and the administration of follow-up clinical breast examination to improve collective knowledge of prognosis, survival, and relative success of various therapies, such as the National Cancer Grid External Quality Assurance Scheme (EQAS) for histopathology^d^	Standardize pathology collection of biopsy and tumor tissue and reorient biopsy fixation to improve diagnostic accuracy and treatment Support pregnant woman to reduce carcinogenic exposures during key window of susceptibility for mother and child	

*BC* breast cancer, *TNBC* triple-negative breast cancer

^a^National Health Portal (NHP) [26]

^b^Tanwar et al. [27]

^c^National Cancer Grid, Collaboration for Cancer Care [28]

^d^National Cancer Grid, Collaboration for Cancer Care [29]

### ***Summary of recommendations (Table ******1******)***

Recommendations included de novo efforts as well as building on the success of existing infrastructure or efforts [25]. One of the broadest reaching recommendations aimed to reduce stigma around what it means to be a female in Indian society. Breast cancer risk reduction and improved early detection and screening demands a better understanding by health care providers and Indian Public Health officials of the barriers women face when considering self-breast examination. One should consider a woman’s comfortability in performing a self-breast examination as well as the more nuanced comfortability of asking questions about the risk of breast cancer or even seeking treatment. Therefore, the recommendation is a multi-level effort to understand and address the core cultural barriers to body awareness that provides programming and resources for improved BC prevention, detection, and treatment at all levels.

### Standardization as a key to moving forward

Another major summary of our discussions included the need to address the disagreement within the medical community about the nature and severity of the problem itself. While attendees agreed that early-onset BC is a major concern, there was much disagreement about accuracy (e.g., have rates increased or has reporting just improved?). This issue underscored the need for improved standardization of molecular subtyping across clinical subtyping in India. Increased standardization and monitoring for data quality as well as pooling of data across regions will help improve monitoring of treatment outcomes. There was agreement that there needs to be a greater implementation of a national uniform standard of care.

## Conclusion

All experts agreed that greater awareness of women’s health and BC is needed across India as well as the fact that more needs to be done to understand hereditary breast cancer in India, as a unique BRCA variant has been described in the Indian population [6]. The higher rate of triple-negative BC observed in India in comparison to other countries may be partially explained by the detection methods in India, but more research and a National Breast Cancer Registry is needed. A call for improved standardization of molecular subtyping across clinical centers in India as well as etiological studies of environmental exposures and BC within existing cohorts and case–control studies will be essential to understand the drivers of early age of onset and the etiology behind increasing BC incidence rates in India.

## Abbreviations

APC: Annual percent change
BC: Breast cancer
BRCA: BReast CAncer gene
DALY: Disability-adjusted life years
EDC: Endocrine disrupting chemicals
PAH: Polycyclic aromatic hydrocarbons
TNBC: Triple-negative breast cancer
US: United States
